# Supplementary material for: Modulation of lytic molecules restrain serial killing in γδ T lymphocytes
Source: Nat Commun. 2023 Sep 27;14:6035. doi: 10.1038/s41467-023-41634-7 (PMC10533871; doi:10.1038/s41467-023-41634-7)
Supplement: Supplementary file 1 — Supplementary Information [file 41467_2023_41634_MOESM1_ESM.pdf]

Supplementary Information File

Modulation of lytic molecules restrain serial killing in γδ T lymphocytes

Authors

Patrick A. Sandoz[1,†,*], Kyra Kuhnigk[2,†], Edina K. Szabo[3,4], Sarah Thunberg[1], Elina Erikson[1], Niklas Sandström[1], Quentin Verron[1], Andreas Brech[5,6,7], Carsten Watzl[8], Arnika K. Wagner[2], Evren Alici[2], Karl-Johan Malmberg[2,3,4], Michael Uhlin[9,10] and Björn Önfelt[1,2,11,*]

Affiliations

[1] Department of Applied Physics, Science for Life Laboratory, KTH Royal Institute of Technology, Stockholm, Sweden

[2] Department of Medicine Huddinge, Karolinska Institutet, Karolinska University Hospital, Stockholm, Sweden.

[3] Precision Immunotherapy Alliance, University of Oslo, Oslo, Norway

[4] Department of Cancer Immunology, Institute for Cancer Research, Oslo University Hospital, Oslo, Norway.

[5] Cancell, Centre for Cancer Cell Reprogramming, Department for Clinical Medicine, University of Oslo, Oslo, Norway

[6] Department of Biosciences, University of Oslo, Oslo, Norway.

[7] Department of Mol. Cell Biology, Institute for Cancer Research, Oslo University Hospital, Oslo, Norway

[8] Department for Immunology, Leibniz Research Centre for Working Environment and Human Factors, TU Dortmund, Dortmund, Germany

[9] CLINTEC, Karolinska Institutet, Stockholm, Sweden

[10] Department of Clinical Immunology and Transfusion Medicine, Karolinska University Hospital, Stockholm, Sweden

[11] Department of Microbiology, Tumour and Cell Biology, Karolinska Institutet, Stockholm, Sweden

[†] Equally Contributing Authors

* Corresponding Authors

Corresponding authors email: psandoz@kth.se & onfelt@kth.se

Supplementary Figures

Supplementary Figure 1.

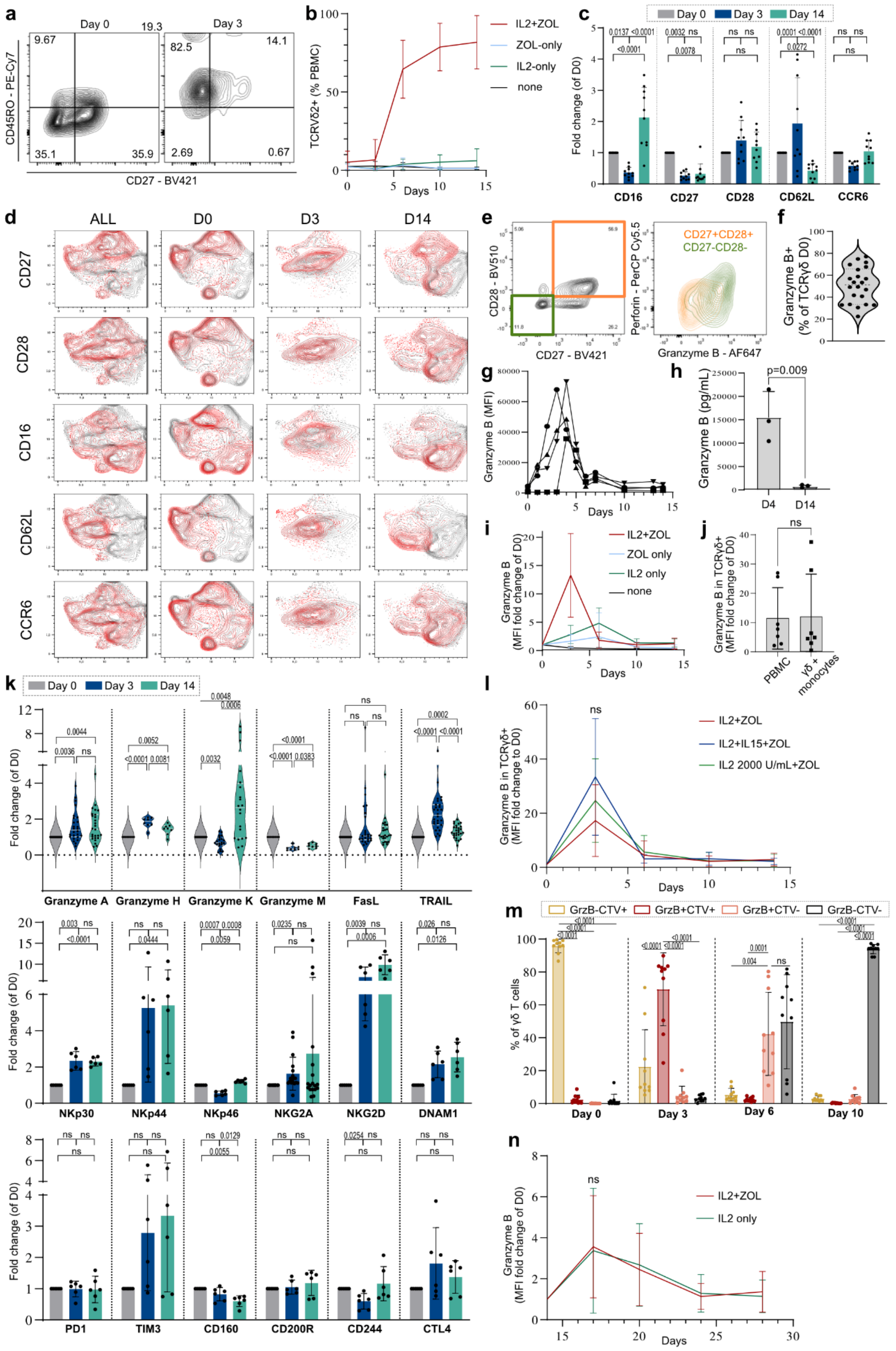

Supplementary Figure 1. Vγ9Vδ2 T cells transiently upregulate granzyme B before expansion. Dynamics of Vγ9Vδ2 T cells expansion in peripheral blood mononuclear cells (PBMC) stimulated with zoledronate (ZOL) and IL2 were measured by flow cytometry at the indicated days. (a) CD45RO & CD27 expression in Vγ9Vδ2 T cells. (b) Percentage of Vδ2 T cells in PBMC expansion upon IL2+ZOL, IL2-only, ZOL-only or none, n=3-12 donors. (c) Statistical analysis (ANOVA) of the markers on the UMAP clustering of Fig. 1e-f, n=10 donors. One dot represents the measurement for one donor. (d) Individual distribution of the markers shown on the UMAP graphs of Fig. 1e-f. (e) Gating on CD27+CD28+ or CD27-CD28- (left) in Vγ9Vδ2 T cells and corresponding granzyme B and perforin expression (right) (10 concatenated donors). (f) Percentage of granzyme B positive resting Vγ9Vδ2 T cells for n = 22 donors. (g) Daily measurement of granzyme B expression in Vγ9Vδ2 T cells during the expansion for n=4 donors. (h) ELISA of granzyme B in lysed γδD4 or γδD14, n=3 donors. (i) Granzyme B expression in Vγ9Vδ2 T cells from PBMCs stimulated with IL2+ZOL, IL2-only, ZOL-only or none, n=6 donors. (j) Granzyme B expression in γδ T cells on day 3 from either PBMCs or isolated γδ T cells co-cultured with isolated autologous monocytes, and stimulated with IL2+ZOL, n=7 donors. (k) Top-row, violin plot (centre line, median; dotted lines, upper and lower quartiles) and statistical comparison of fold changes according to Fig. 1g, n=8-28 donors. Middle and bottom rows, statistical analysis of the activating / inhibitory receptors and exhaustion markers of Fig. 1g, analysis by one-way ANOVA. (l) Granzyme B expression fold change (normalized to initial level) in Vγ9Vδ2 T cells of PBMC stimulated with IL2+ZOL with or without IL15 addition, n=11 donors, or with the addition or not of IL2 at 2000 IU, n=7 donors. (m) Proportions of granzyme B and CTV expressed at high levels in γδ T cells (as of Fig. 1k) at the indicated timepoints of the expansion, n=10 donors, analysis by one-way ANOVA. (n) 14-days expanded γδ T cells were stained with CTV and re-stimulated in autologous thawed PBMCs with IL2 only or with IL2+ZOL. Granzyme B protein level was measured by flow cytometry in the CTV+ cells at the indicated timepoints, n=9 donors. All applicable plots show means with standard deviations unless specifically stated otherwise. Statistics were calculated using two-tailed paired t test if not stated otherwise. ns: not-significant.

Supplementary Figure 2.

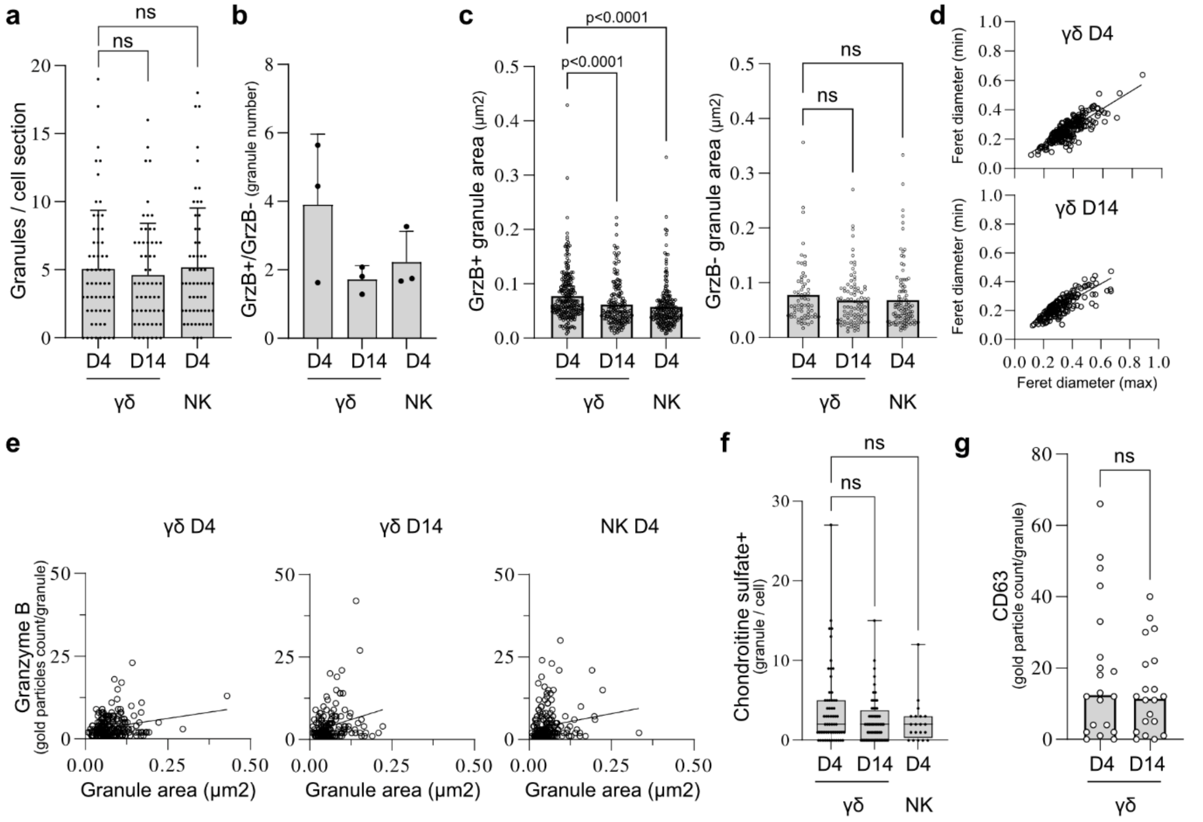

Supplementary Figure 2. Vγ9Vδ2 T cell lytic vesicles are mature and loaded with granzyme B. Immuno-EM gold nanoparticle labelling with anti-granzyme B (a-e) or anti-chondroitin sulfate (f) or anti-CD63 (g) antibodies of sections from γδD4 or γδD14 T cells or NKD4 cells from the same donors. 20 cells from the cell sections were imaged by transmission electron microscopy for each donor. (a) Quantification of the average number of dense core granules per cell (one transversal section per cell), means with standard deviations. One dot represents the average number of dense core granules for one cell section. (b) Ratio between the number of granzyme B labelled granules and the granzyme B absent granules, means with standard deviations. (c) Area of granzyme B unlabelled granules (right) and granzyme B labelled granules (left), bar plots represent the medians. (d) Minimum and maximum Feret diameters for each granule (circles) and the linear regression (black line) for γδ D4 or γδ D14 T cells. (e) Comparison of the granzyme B gold particle count per granule and the corresponding granule area (circles) and the linear regression (black line). (f) Number of anti-chondroitin sulfate labelled granules per cell, means with standard deviations. (g) Anti-CD63 labelling of 4-days or 14-days expanded γδ T cells of the same donor. Counts were pooled from 3 donors (a-e) or 1-3 donors (f-g). All analysis by Kruskal-Wallis test, ns: not-significant.

Supplementary Figure 3.

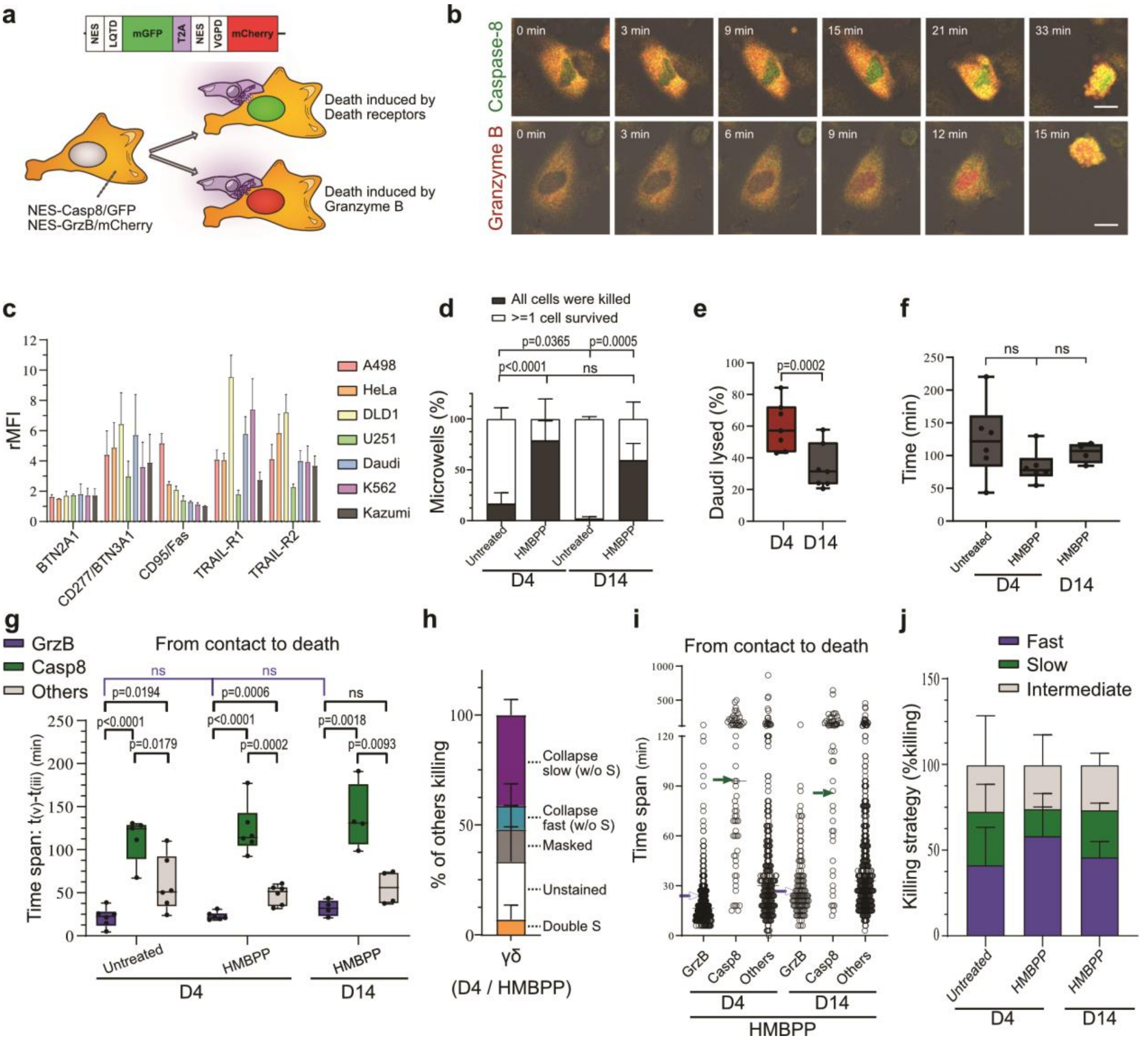

Supplementary Figure 3. Individual Vγ9Vδ2 T cell killing potential and cytotoxic strategies. (a) Scheme of the dual fluorescent reporter structure and function. Construct was inserted in the lentivirus vector. NES: nuclear exclusion signal. LQTD: specific for caspase-8 cleavage (downstream of death receptors activation). VGPD: specific for granzyme B cleavage. Death receptors-mediated killing leads to the diffusion of the green fluorescent protein into the nucleus. Granzyme-B mediated killing leads to the diffusion of the mCherry red fluorescent protein into the nucleus. (b) Examples of the two killing strategies as observed during the on-chip killing assay with isolated γδ T cells against A498GBDR target cells: death receptors mediated killing (top); granzyme B mediated killing (bottom). Scale bars: 10 µm. Time starts at the first γδ T cell – target contact. (c) Expression of CD95/FasL, TRAIL-R2, CD277/BTN3A and BTN2A1 in various cell lines as measured by flow cytometry. rMFI shows the MFI measurement of the stained sample divided by its unstained control, n=2-6 replicates. (d) Fraction of the wells in which all A498GBDR targets were killed by γδ T cells during the 16 hr on-chip killing assay, n=4-6 donors. (e) Proportion of Daudi target cells killed by isolated γδD4 or γδD14 T cells at a 1:1 E:T ratio as measured by flow cytometry from in-tube 4 hr co-cultures, n=7 donors. The box limits represent upper and lower quartiles, and whiskers indicate the minimum and maximum values. (f) Time between the start of the on-chip killing assay and the first interaction between a γδ T cell and a A498GBDR for a lethal contact. n=6 (γδD4) and 4 (γδD14) donors. (g) Killing dynamics: time between the first contact of a γδ T cell with its target and the target's death for each mechanism, n=4-6 donors. (h) Others killing events: contribution of double nuclear staining (Double S), insufficiently stained A498GBDR, masked A498GBDR during killing, fast A498GBDR collapse without nuclear staining (w/o S) or slow A498GBDR collapse without staining in the on-chip killing assay with γδD4 T cells and A498GBDR upon HMBPP, n=4 donors. (i) Killing dynamics for individual events (first cell-cell contact to target cell death) for each mechanism in the on-chip killing assay using γδD4 or γδD14 T cells. The blue arrows show the respective upper quartile (75%) of granzyme-B mediated killing dynamics. The green arrows show the respective lower quartile of the death-receptors mediated killing dynamics, n=4-6 donors pooled. (j) Proportion of the killing strategies (overall killing) after reclassification of the other mechanisms using the thresholds indicated in (i). All graphs show means with standard deviations unless specifically stated otherwise. The box plot line shows the median, the limits represent upper and lower quartiles, and whiskers indicate the minimum and maximum values. (e-g) One dot represents the measurement for one donor. Statistics were calculated using two-tailed Student's t test, ns: not-significant.

Supplementary Figure 4.

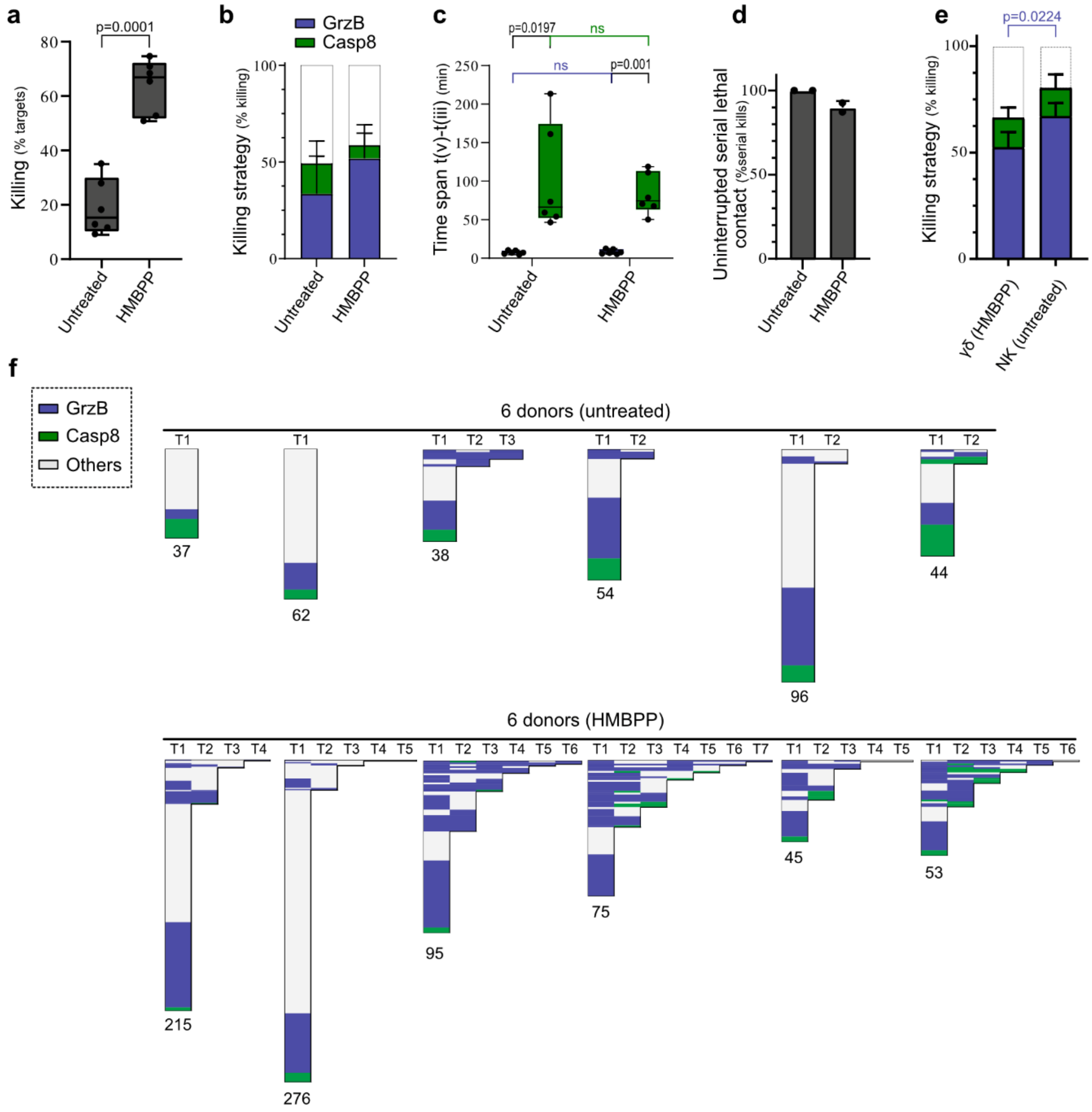

Supplementary Figure 4. Characterization of Vγ9Vδ2 T cells serial killing dynamics. On-chip killing assay of A498GBDR target cell killing by γδD4 T cells in 350 μm wells with or without 1.5 μM HMBPP added at the start of the assay, n=6 donors. (a) Proportion of A498GBDR target cells killed (b) Proportion of the killing strategies analysed by assessing the diffusion of the reporter into nuclei. (c) Time span between the first detection of reporter signal in the nucleus and the target death. (d) Proportion of killing events that was immediately followed by another killing event during serial killing. For this, we evaluated the contacts made by serial killing T cells from the first killing event to the last killing event for 2 donors. During active serial killing most target cells were killed and very rarely did the cell alternate between killing and non-killing. However, after the last observed killing event the T cells could form several non-lethal contacts with target cells. (e) Proportion of the killing strategies used by γδD4 T cells (1.5 μm HMBPP) or NKD4 cells, n=4 donors. (f) Killing series for individual donors, numbers under the first column indicate the number of one-target killed events (donors are pooled in Fig. 3c-d). All applicable graphs show means with standard deviations unless specifically stated otherwise. The box plot line shows the median, the limits represent upper and lower quartiles, and whiskers indicate the minimum and maximum values. (a,c-d) One dot represents the measurement for one donor. Statistics were calculated using two-tailed paired t-test, ns: not-significant.

Supplementary Figure 5.

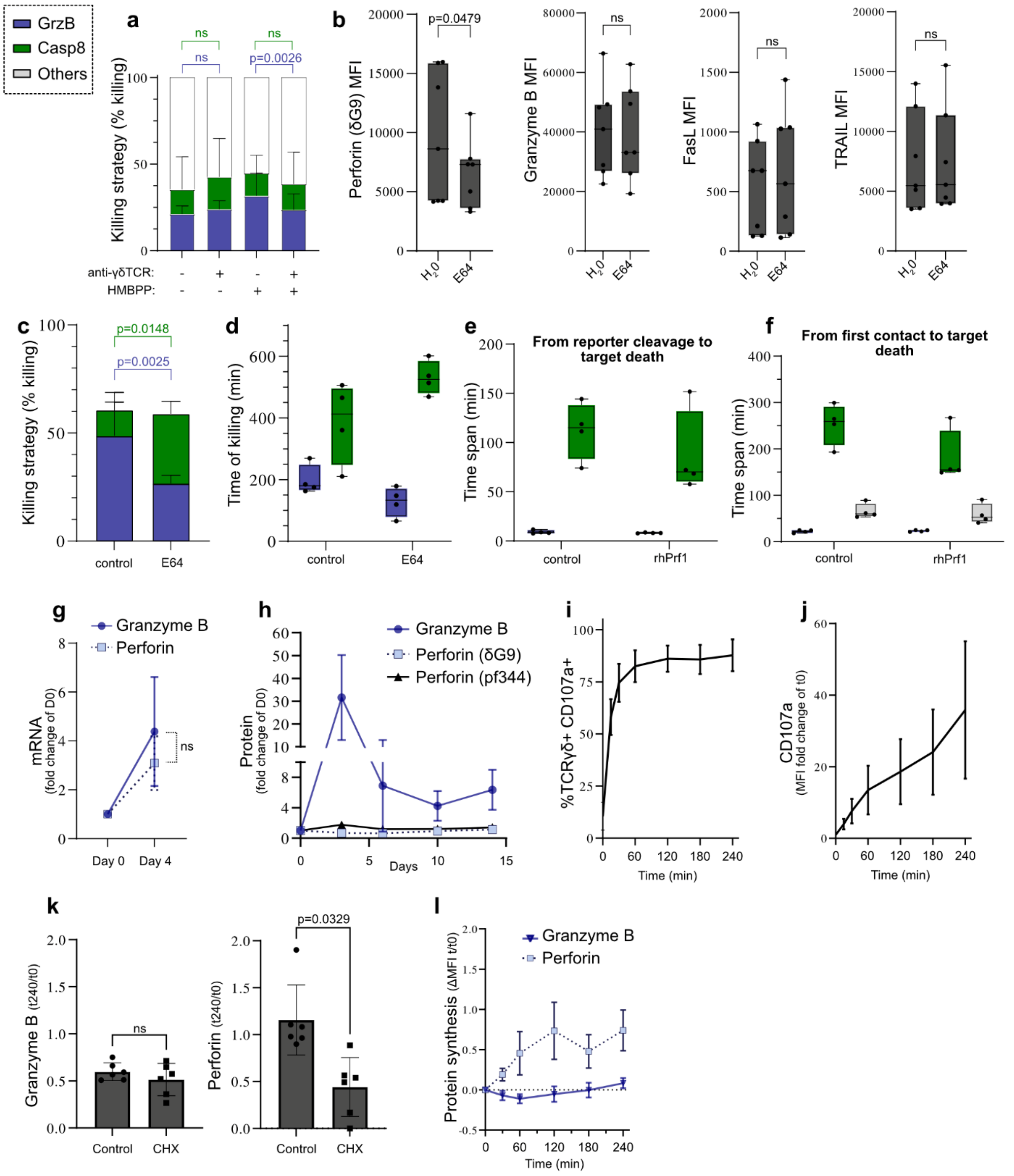

Supplementary Figure 5. Perforin availability is a bottleneck for γδ T cell serial killing (a) Proportion of the killing strategies upon blocking with 5 μg/mL of anti-γδTCR (or IgG antibody control) and treatment with or without 1.5 μM HMBPP, n=4 donors. (b-d) Isolated γδD4 T cells were treated or not with the E64 inhibitor for 24 hr. (b) Expression levels of perforin (δG9 clone: mature perforin), granzyme B, TRAIL and FasL measured by flow cytometry, n=7 donors. The box limits represent upper and lower quartiles, and whiskers indicate the minimum and maximum values. (c) On-chip killing assay with γδD4 T cells with or without E64 pre-treatment against A498GBDR target cells treated with 1.5 μM of HMBPP. Proportion of the killing strategies and (d) Time of killing from the start of the assay for the different strategies with or without E64 treatment, n=4 donors. (e-f) On-chip killing assay with γδD4 T cells against A498GBDR target cells treated with 1.5 μM of HMBPP, with or without the addition of 60 ng/mL recombinant human perforin (rhPrf1). (e) Time between the first detection of reporter signal in the nucleus until the target death, n=4 donors. (f) Time between the first contact of a γδ T cell with its target and target cell death for each killing mechanism, n=4 donors. (g) mRNA levels for granzyme B and perforin in resting γδ T cells compared to γδD4 T cells as measured by qPCR after normalization to a housekeeping gene, n=4 donors. (h) Granzyme B and perforin (precursor and mature: clone Pf-344 or mature: clone δG9) protein levels were measured by flow cytometry at the indicated timepoints and normalized to their respective initial levels, n=3 donors. (i) In-tube CD107a degranulation assay of γδD4 T cells with 1.5 μM HMBPP as measured by flow cytometry at the indicated timepoints, n=3 donors. (j) In-tube CD107a degranulation assay of γδD4 T cells with 1.5 μM HMBPP and monensin (blocking the surface recycling of CD107a) as measured by flow cytometry at the indicated timepoints, n=3 donors. (k) Granzyme B (left) or perforin (right) cellular content measured by flow cytometry at the terminal timepoint (240 min) in γδD4 T cells treated with 1.5 μM HMBPP with or without 2 μg/mL of cycloheximide (CHX) from the start of degranulation assay, n=6 donors. (l) Newly expressed granzyme B and perforin as the difference between untreated and cycloheximide-treated (data from Fig. 5i) γδD4 T cells during killing assay. All applicable graphs show means with standard deviations unless specifically stated otherwise. The box plot line shows the median, the limits represent upper and lower quartiles, and whiskers indicate the minimum and maximum values. (b,d-f,k) One dot represents the measurement for one donor. Statistics were calculated using two-tailed paired t test, ns: not-significant.

Supplementary Figure 6.

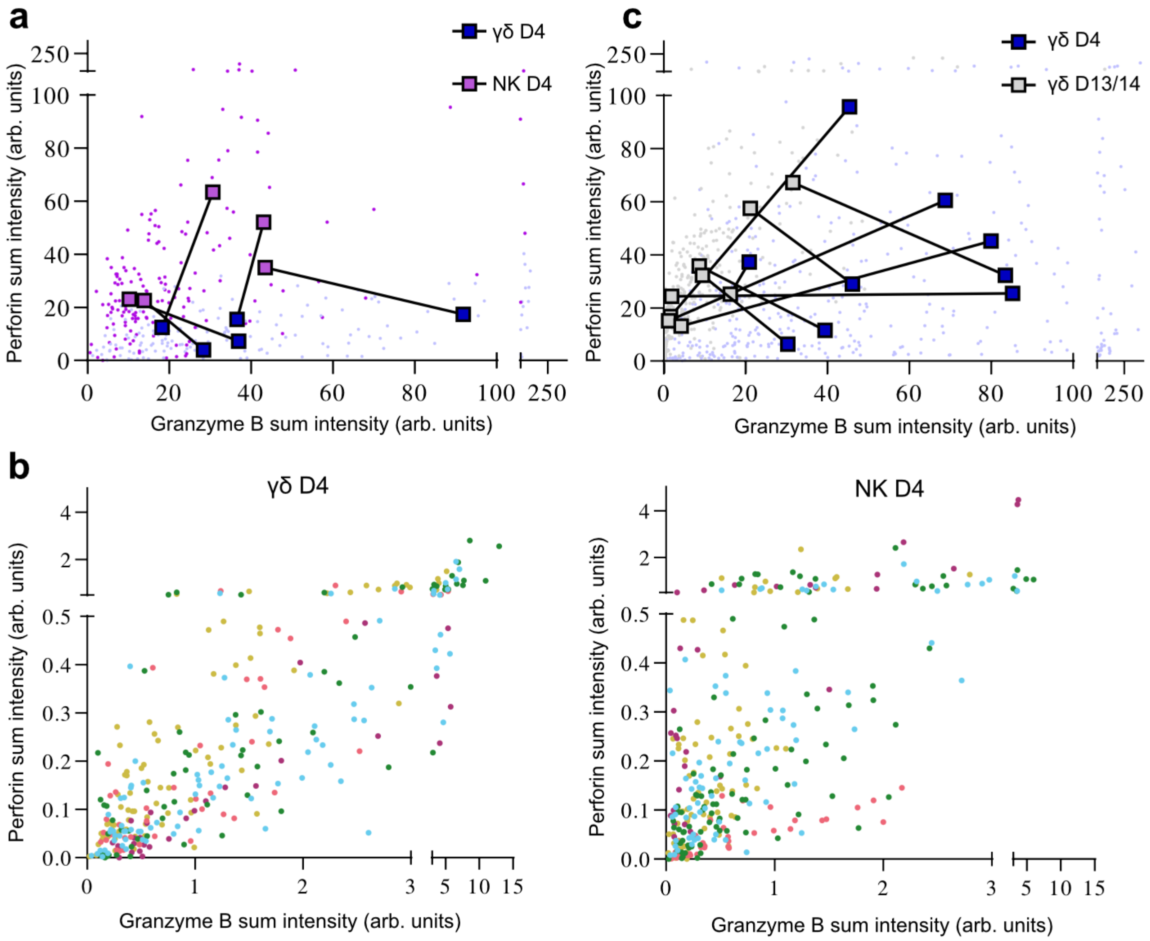

Supplementary Figure 6. Vγ9Vδ2 T cells segregate granzyme B from perforin in different lytic vesicles (a) Granzyme B and perforin signal sum intensity in CD107a+ vesicles of γδD4 T cells and NKD4 cells (black lines connect cell types from the same donors). The square boxes are the means for each donor (n=5 donors, each ≥15 cells). To compensate for experimental fluctuations between experiments, the sum intensity of each object is normalized to the standard deviation of all objects measured from the condition (cell type) of that experiment. (b) Sum intensity of granzyme B versus perforin in all CD107a+ lytic vesicles of 5 representative cells for γδD4 (left) or NKD4 (right). (c) Granzyme B and perforin signal mean intensity in CD107a+ vesicles of γδD4 or γδD13/14 T cells. The square boxes are the means for each donor (n=9 donors, each ≥15 cells). Lines connect cell types from the same donors. To compensate for experimental fluctuations between experiments, the sum intensity of each object is normalized to the standard deviation of all objects measured from the condition of that experiment.

Supplementary Figure 7.

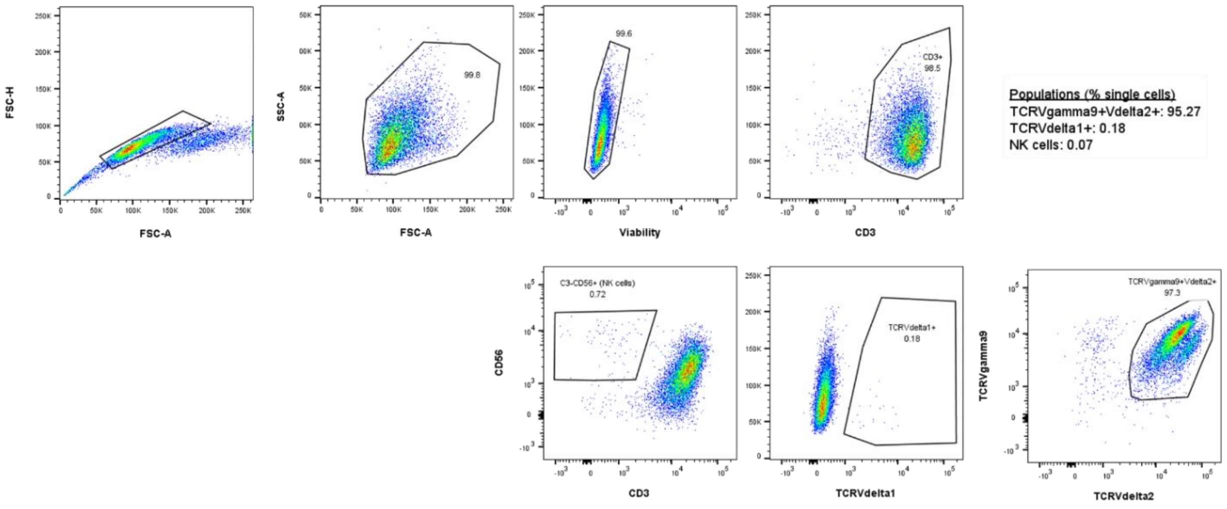

Supplementary Figure 7. Example of the gating strategy to assess the purity of Vγ9Vδ2 T cells (CD3+TCRVγ9+TCRVδ2+) after isolation as compared to NK (CD3-CD56+) and Vδ1+ cells (CD3+TCRVδ1+).

Supplementary Table 1

Antigen	Clone	Fluorophore	Supplier	Code	RRID	Dilution
Actin/Phalloidin		Alex Fluor 568	Thermo Fisher	A12380		1:10000
BTN2A1			Sigma-Aldrich	HPA019208	AB_1845492	1:100
CCR6	11A9	PE-Cy7	BD Pharmingen	560620	AB_1727440	1:40
CCR7	150503	PE	BD Pharmingen	560765	AB_2033949	1:20
CD107a	H4A3	Biotin	BD Pharmingen	555799	AB_396133	1:20
CD107a	H4A3	BV421	BD Horizon	562623	AB_2737685	1:100
CD107a	H4A3	Alex Fluor 647	BD Pharmingen	562622	AB_2737684	1:100
CD16	3G8	APC	BD Pharmingen	561248	AB_10612010	1:50
CD16	3G8	BV605	BD Pharmingen	563172	AB_2744297	1:50
CD160	BY55	FITC	Invitrogen	MA5-16600	AB_2538099	1:40
CD19	SJ25C1	PE-Cy7	BD Pharmingen	557835	AB_396893	1:40
CD200R	OX108	PE	Invitrogen	12-9201-42	AB_10717665	1:40
CD223/Lag3	11C3C65	PE	BioLegend	369306	AB_2629592	1:40
CD244	eBioDM244	APC	Invitrogen	17-5837-42	AB_10670227	1:40
CD253/TRAIL	RIK-2	BV421	BD Horizon	564243	AB_2738696	1:50
CD261/TRAIL-R1	S35-934	PE	BD Pharmingen	564180	AB_2738648	1:40
CD262/TRAIL-R2	YM366	PE	BD Pharmingen	565499	AB_2732871	1:40
CD27	M-T271	BV421	BD Horizon	562513	AB_11153497	1:50
CD277/BTN3A1	232-5	BV605	BD OptiBuild	742828	AB_2741080	1:50
CD277/BTN3A1	20.1, BT3.1		Invitrogen	14-2779-82	AB_467550	1:50
CD28	CD28.2	BV510	BioLegend	302936	AB_2562030	1:100
CD3	UCHT1	Alexa Fluor 700	BD Pharmingen	557943	AB_396952	1:40
CD45RA	HI100	BV605	BD Horizon	562886	AB_2737865	1:50
CD45RO	UCHL1	PE-Cy7	BD Pharmingen	560608	AB_1727499	1:50
CD56	B159	PerCP-Cy5.5	BD Pharmingen	560842	AB_2033964	1:50
CD62L	DREG-56	PE	BD Pharmingen	555544	AB_395928	1:20
CD63	H5C6-s		DSHB		AB_528158	1:50
CD8	SK1	APC-H7	BD Pharmingen	560179	AB_1645481	1:40
CD95/Fas	DX2	BV421	BD Horizon	562616	AB_2737679	1:40
CX3CR1	2A9-1	BV605	BD OptiBuild	744488	AB_2742268	1:40
CD178/FasL	NOK-1	APC	BD Pharmingen	564262	AB_2738714	1:50
CD226/DNAM1	DX11	BV605	BD OptiBuild	742495	AB_2740828	1:40
Chondroitin Sulfate 4	2B6		AMSBIO	270432	AB_10891938	1:10
CTLA4	BNI3	BV421	BD Horizon	562743	AB_2737762	1:40
Granulysin	RB1	Alexa Fluor 488	BD Pharmingen	558254	AB_2869123	1:10
Granzyme A	GzA-3G8.5	PE	Invitrogen	12-5831-82	AB_2572631	1:40
Granzyme B	GB11	Alexa Fluor 647	BD Pharmingen	561999	AB_10897997	1:40
Granzyme B	496B		Invitrogen	14-8889-82	AB_2572909	1:10
Granzyme H	P20718	Alexa Fluor 488	G Biosciences	ITA2121		1:200
Granzyme K	G3H69	Alexa Fluor 647	BD Pharmingen	566655	AB_2869812	1:40
Granzyme M	4B2G4	eFluor660	Invitrogen	50-9774-42	AB_2574374	1:100
NKG2A	131411	BV605	BD OptiBuild	747921	AB_2872382	1:40
NKG2C	REA205	Vio Bright FITC	Miltenyi Biotech	130-117-707	AB_2728023	1:40
NKG2D	1D11	PerCP-Cy5.5	BD Pharmingen	562364	AB_11154225	1:40
Nkp30	p30-15	BV510	BD OptiBuild	743170	AB_2741321	1:40
Nkp44	p44-8	BV421	BD Pharmingen	744299	AB_2742129	1:40
Nkp46	9E2	PE-Cy7	BD Pharmingen	562101	AB_10894195	1:40
PD1	EH12.1	PE-Cy7	BD Pharmingen	561272	AB_10611585	1:40
Perforin	dG9	Pacific Blue	BioLegend	308118	AB_10899565	1:50
Perforin	dG9	Alexa Fluor 488	BioLegend	308108	AB_493252	1:20
Perforin	dG9	PerCP-Cy5.5	BD Pharmingen	563762	AB_2738409	1:40
Perforin	Pf-344	FITC	Mabtech	3465-7	AB_1925742	1:40
Perforin	Pf-344	PF647P	Mabtech	3465-72-100T	AB_2888642	1:40
Streptavidin		Alexa Fluor 568	Invitogen	S11226	AB_2315774	1:500
TCR gd pan	11F2	PE	Miltenyi Biotech	130-113-504	AB_2733905	1:100
TCR gd pan	IMMU510		Beckman Coulter	IM1349	AB_131619	1:50
TCR Vd1	TS8.2	FITC	Invitrogen	TCR2730	AB_223624	1:20
TCR Vd2	B6	PE	BioLegend	331408	AB_1089232	1:40
TCR Vd2	B6	APC/Cy7	BioLegend	331439	AB_2860864	1:40
TCR Vg9	B3	FITC	BioLegend	331306	AB_1236403	1:40
TCR Vg9	B3	PE-Cy7	BioLegend	331320	AB_2814209	1:40
Tigit	A15153G	Alexa Fluor 647	BioLegend	372724	AB_2715972	1:40
TIM3	7D3	BV605	BD OptiBuild	742856	AB_2744030	1:40
Viability stain 510			BD Horizon	564406	AB_2869572	1:1000
Viability stain 700			BD Horizon	564997	AB_2869637	1:1000

Supplementary Table 1 Legend

List of the antibodies/staining used in this study.
