# Supplementary material for: Modulation of lytic molecules restrain serial killing in γδ T lymphocytes
Source: Nat Commun. 2023 Sep 27;14:6035. doi: 10.1038/s41467-023-41634-7 (PMC10533871; doi:10.1038/s41467-023-41634-7)
Supplement: Supplementary file 3 — Description of Additional Supplementary Files [file 41467_2023_41634_MOESM3_ESM.pdf]

Description of Additional Supplementary Files

File Name: Supplementary Movie 1
Description: **Example of γδ T cell mediated death through granzyme B (A) or death receptors (B, start at 11 sec).** A498^{GBDR} target cell stably expressing the dual fluorescent reporter NES-ELQTD-GFP-T2A-NES-VGPD-mCherry co-cultured with unlabelled γδ T cells. Under attack of a γδ T cell, the fluorescent reporter is cleaved and mCherry (granzyme B-mediated death (A) or GFP (death receptor-mediated death through caspase 8 cleavage (B) can diffuse into the nucleus. Frame rates are slowed down to highlight nuclear staining.

File Name: Supplementary Movie 2
Description: **Examples for individual outcomes of γδ T cells co-cultured with A498^{GBDR} target cells.** (A) γδ T cell migrating, dividing (timepoint 11 sec), and contacting A498 target cells during a 16-hour assay, without being able to kill the target cell. (B, start at 21 sec) Consecutive kills by a γδ T cell. After three kills the γδ T cell divides (timepoint 42 sec) before continuing to kill the fourth target. (C, start at 53 sec) γδ T cell kills one target cell, keeps on migrating and dividing but is not able to mediate a second kill. Frame rates are slowed down to highlight nuclear staining and cell division.

File Name: Supplementary Movie 3
Description: **Characterization of unidentified deaths.** (A) Both GFP and mCherry, are cleaved and diffuse into the nucleus, resulting in a double positive death. (B, start at 15 sec) The target cell starts shrinking with no visible signal in the nucleus and then collapses over a long period. (C, start at 32 sec) The target cell shrinks and collapses quickly in 2 frames (or less) without a visible nuclei signal. Frame rates are slowed down to highlight nuclear staining and collapsing of the target cells.

File Name: Supplementary Movie 4
Description: **Addition of 15 µm recombinant granulysin to A498^{GBDR}.** With the addition of granulysin, target cells are collapsing slowly. The fluorescent reporter can diffuse into the nucleus indicating nuclear envelope rupture.

File Name: Supplementary Movie 5
Description: **Killing events by γδ T cells can be mediated through committed (A) or uncommitted (B, start at 11 sec) contacts.** (A) γδ T cell contacts the A498 target and converges toward the contact site forming a committed contact that lasts longer than 2 imaging frames. (B) γδ T cell is interacting with the target cell while continue to migrate. While no long contacts or commitment to the target are observed, the target is still killed. Frame rates are slowed down to highlight contact of γδ T cell and target cell.

File Name: Supplementary Movie 6
Description: **γδ T cells without target cells in the 80 µm well platform.** (A) γδ T cells are viable, migrating and proliferating inside the 80 µm microwells over a 16-hour assay. (B, start at 18 sec) Treatment with HMBPP can lead to fratricide if γδ T cell are in close contact. Frame rate is slowed down to highlight fratricide.

File Name: Supplementary Movie 7
Description: **γδ T cells co-cultured with A498^{GBDR} target cells in 350 µm wells show limited serial killing potential.** γδ T cell No. 1 (red) kills one target through death receptor-mediated death by cleaving caspase 8 (event E). γδ T cells No. 4 (cyan) and 10 (blue) are contacting targets together and kill two consecutive targets through granzyme B mediated death (event A and B). γδ T cell No. 8 (purple) kills two consecutive targets through granzyme B mediated death (event C and D). All remaining γδ T cells fail to induce target cell death. Frame rates are slowed down to highlight contact of γδ T cell, nuclear staining and target death.
