# Supplementary material for: Modulation of lytic molecules restrain serial killing in γδ T lymphocytes
Source: Nat Commun. 2023 Sep 27;14:6035. doi: 10.1038/s41467-023-41634-7 (PMC10533871; doi:10.1038/s41467-023-41634-7)
Supplement: Supplementary file 11 — Reporting Summary [file 41467_2023_41634_MOESM11_ESM.pdf]

nature portfolio

Corresponding author(s): Björn Önfelt

Last updated by author(s): August 23, 2023

Reporting Summary

Nature Portfolio wishes to improve the reproducibility of the work that we publish. This form provides structure for consistency and transparency in reporting. For further information on Nature Portfolio policies, see our Editorial Policies and the Editorial Policy Checklist.

Statistics

For all statistical analyses, confirm that the following items are present in the figure legend, table legend, main text, or Methods section.

n/a	Confirmed	
☐	☒	The exact sample size (*n*) for each experimental group/condition, given as a discrete number and unit of measurement
☐	☒	A statement on whether measurements were taken from distinct samples or whether the same sample was measured repeatedly
☐	☒	The statistical test(s) used AND whether they are one- or two-sided<br>*Only common tests should be described solely by name; describe more complex techniques in the Methods section.*
☒	☐	A description of all covariates tested
☒	☐	A description of any assumptions or corrections, such as tests of normality and adjustment for multiple comparisons
☐	☒	A full description of the statistical parameters including central tendency (e.g. means) or other basic estimates (e.g. regression coefficient) AND variation (e.g. standard deviation) or associated estimates of uncertainty (e.g. confidence intervals)
☐	☒	For null hypothesis testing, the test statistic (e.g. *F*, *t*, *r*) with confidence intervals, effect sizes, degrees of freedom and *P* value noted<br>*Give P values as exact values whenever suitable.*
☒	☐	For Bayesian analysis, information on the choice of priors and Markov chain Monte Carlo settings
☒	☐	For hierarchical and complex designs, identification of the appropriate level for tests and full reporting of outcomes
☒	☐	Estimates of effect sizes (e.g. Cohen's *d*, Pearson's *r*), indicating how they were calculated

Our web collection on statistics for biologists contains articles on many of the points above.

Software and code

Policy information about availability of computer code

Data collection	Airyscan confocal images were acquired using Zen software (Carl Zeiss) version 2.3 SP1. Widefield time-lapse microscopy was acquired using either Zen software (Carl Zeiss) version 3.1 (ZEN PRO) or 2 (blue version). Flow cytometry data was acquired using BD FACS Diva software version 9.0.
Data analysis	Images were analysed using ImageJ (NIH) fiji-win64x version and Imaris software (Oxford Instruments) x64 version 9.5.1. Flow cytometry data was analysed using FlowJo (BD) version 10.7.1.

For manuscripts utilizing custom algorithms or software that are central to the research but not yet described in published literature, software must be made available to editors and reviewers. We strongly encourage code deposition in a community repository (e.g. GitHub). See the Nature Portfolio guidelines for submitting code & software for further information.

Data

Policy information about availability of data

All manuscripts must include a data availability statement. This statement should provide the following information, where applicable:

- Accession codes, unique identifiers, or web links for publicly available datasets
- A description of any restrictions on data availability
- For clinical datasets or third party data, please ensure that the statement adheres to our policy

The data that support the findings of this study are available from the corresponding author upon reasonable request.

Research involving human participants, their data, or biological material

Policy information about studies with human participants or human data. See also policy information about sex, gender (identity/presentation), and sexual orientation and race, ethnicity and racism.

Reporting on sex and gender	Anonymous buffy coats from healthy human donors were obtained from the Karolinska Hospital (Sweden) and used to isolate specific immune cells for this study. This requires no ethical permit according to the local regulations. No sex or gender information is provided with the buffy coats.
Reporting on race, ethnicity, or other socially relevant groupings	No race, ethnicity or other socially relevant groupings information is provided with the buffy coats.
Population characteristics	The cohort consists of healthy donors, no covariates are included.
Recruitment	The anonymous buffy coats from healthy donors were obtained from the department of Klinisk immunologi och transfusionsmedicin in Karolinska Hospital, Huddinge, Sweden.
Ethics oversight	Not applicable

Note that full information on the approval of the study protocol must also be provided in the manuscript.

Field-specific reporting

Please select the one below that is the best fit for your research. If you are not sure, read the appropriate sections before making your selection.

☒ Life sciences ☐ Behavioural & social sciences ☐ Ecological, evolutionary & environmental sciences

For a reference copy of the document with all sections, see nature.com/documents/nr-reporting-summary-flat.pdf

Life sciences study design

All studies must disclose on these points even when the disclosure is negative.

Sample size	For electron microscopy and for immunofluorescence Airyscan microscopy, at least 20 cells were imaged at high resolution for each donor. For flow cytometry, at least 6 donors for each marker were measured and analysed. The live-cell killing assays were performed for at least 3 donors per condition tested. For these assays, the behaviour of at least 100 gamma delta T effector cells aimed to be manually analysed per condition. For a few experiments, this number was not reached (e.g. 65 or 80 cells available). In such case, more experiments were conducted with new donors for all conditions and added to the dataset (n > 3). The sample size was determined without statistical methods but based on previous publications in the field.
Data exclusions	No technically successful experiment was excluded. Experiments with technical issues (e.g. loss of focus during imaging) were not analysed. In figure 3f,g,i,j: one condition is not provided (events in untreated cells - D14) due to the very low occurrence of these events which prevents any statistical analysis. This exclusion is stated in the text of the manuscript (lines 234-236).
Replication	At least three independent experiments were performed for each of the assays in the manuscript. No technically successful replicate was excluded.
Randomization	The assigned compartment in the microwell chip for each condition was alternated between the replicates assuring randomization of the conditions.
Blinding	Electron microscopy lytic granules analysis was blind. The cells were prepared by the first authors in Stockholm and analyzed by another author in Oslo without knowing the corresponding conditions. Immunofluorescence Airyscan microscopy of granzyme B and perforin was analysed automatically. The image datasets of the live-cell killing assays were randomly assigned to two reseachers and independently analysed based on defined criteria and supported by automatic signal intensity measurements.

Reporting for specific materials, systems and methods

We require information from authors about some types of materials, experimental systems and methods used in many studies. Here, indicate whether each material, system or method listed is relevant to your study. If you are not sure if a list item applies to your research, read the appropriate section before selecting a response.

Materials & experimental systems

n/a	Involved in the study	
☐	☒	Antibodies
☐	☒	Eukaryotic cell lines
☒	☐	Palaeontology and archaeology
☒	☐	Animals and other organisms
☒	☐	Clinical data
☒	☐	Dual use research of concern
☒	☐	Plants

Methods

n/a	Involved in the study	
☒	☐	ChIP-seq
☐	☒	Flow cytometry
☒	☐	MRI-based neuroimaging

Antibodies

Antibodies used

Antibodies used in this study (as in Supplementary Table 1):
(Antigen Clone Fluorophore Supplier Code RRID)
Actin/Phalloidin Alex Fluor 568 Thermo Fisher A12380
BTN2A1   Sigma-Aldrich HPA019208 AB_1845492
CCR6 11A9 PE-Cy7 BD Pharmingen 560620 AB_1727440
CCR7 150503 PE BD Pharmingen 560765 AB_2033949
CD107a H4A3 Biotin BD Pharmingen 555799 AB_396133
CD107a H4A3 BV421 BD Horizon 562623 AB_2737685
CD107a H4A3 Alex Fluor 647 BD Pharmingen 562622 AB_2737684
CD16 3G8 APC BD Pharmingen 561248 AB_10612010
CD16 3G8 BV605 BD Pharmingen 563172 AB_2744297
CD160 BY55 FITC Invitrogen MA5-16600 AB_2538099
CD19 SJ25C1 PE-Cy7 BD Pharmingen 557835 AB_396893
CD200R OX108 PE Invitrogen 12-9201-42 AB_10717665
CD223/Lag3 11C3C65 PE BioLegend 369306 AB_2629592
CD244 eBioDM244 APC Invitrogen 17-5837-42 AB_10670227
CD253/TRAIL RIK-2 BV421 BD Horizon 564243 AB_2738696
CD261/TRAIL-R1 S35-934 PE BD Pharmingen 564180 AB_2738648
CD262/TRAIL-R2 YM366 PE BD Pharmingen 565499 AB_2732871
CD27 M-T271 BV421 BD Horizon 562513 AB_11153497
CD277/BTN3A1 232-5 BV605 BD OptiBuild 742828 AB_2741080
CD277/BTN3A1 20.1, BT3.1 Invitrogen 14-2779-82 AB_467550
CD28 CD28.2 BV510 BioLegend 302936 AB_2562030
CD3 UCHT1 Alexa Fluor 700 BD Pharmingen 557943 AB_396952
CD45RA HI100 BV605 BD Horizon 562886 AB_2737865
CD45RO UCHL1 PE-Cy7 BD Pharmingen 560608 AB_1727499
CD56 B159 PerCP-Cy5.5 BD Pharmingen 560842 AB_2033964
CD62L DREG-56 PE BD Pharmingen 555544 AB_395928
CD63 H5C6-s DSHB AB_528158
CD8 SK1 APC-H7 BD Pharmingen 560179 AB_1645481
CD95/Fas DX2 BV421 BD Horizon 562616 AB_2737679
CX3CR1 2A9-1 BV605 BD OptiBuild 744488 AB_2742268
CD178/FasL NOK-1 APC BD Pharmingen 564262 AB_2738714
CD226/DNAM1 DX11 BV605 BD OptiBuild 742495 AB_2740828
Chondroitin Sulphate 4 2B6 AMSBIO 270432 AB_10891938
CTLA4 BNI3 BV421 BD Horizon 562743 AB_2737762
Granulysin RB1 Alexa Fluor 488 BD Pharmingen 558254 AB_2869123
Granzyme A GzA-3G8.5 PE Invitrogen 12-5831-82 AB_2572631
Granzyme B GB11 Alexa Fluor 647 BD Pharmingen 561999 AB_10897997
Granzyme B 496B Invitrogen 14-8889-82 AB_2572909
Granzyme H P20718 Alexa Fluor 488 G Biosciences ITA2121
Granzyme K G3H69 Alexa Fluor 647 BD Pharmingen 566655 AB_2869812
Granzyme M 4B2G4 eFluor660 Invitrogen 50-9774-42 AB_2574374
NKG2A 131411 BV605 BD OptiBuild 747921 AB_2872382
NKG2C REA205 Vio Bright FITC Miltenyi Biotech 130-117-707 AB_2728023
NKG2D 1D11 PerCP-Cy5.5 BD Pharmingen 562364 AB_11154225
Nkp30 p30-15 BV510 BD OptiBuild 743170 AB_2741321
Nkp44 p44-8 BV421 BD Pharmingen 744299 AB_2742129
Nkp46 9E2 PE-Cy7 BD Pharmingen 562101 AB_10894195
PD1 EH12.1 PE-Cy7 BD Pharmingen 561272 AB_10611585
Perforin dG9 Pacific Blue BioLegend 308118 AB_10899565
Perforin dG9 Alexa Fluor 488 BioLegend 308108 AB_493252
Perforin dG9 PerCP-Cy5.5 BD Pharmingen 563762 AB_2738409
Perforin Pf-344 FITC Mabtech 3465-7 AB_1925742
Perforin Pf-344 PF647P Mabtech 3465-72-100T AB_2888642
Streptavidin Alexa Fluor 568 Invitogen S11226 AB_2315774
TCR gd pan 11F2 PE Miltenyi Biotech 130-113-504 AB_2733905
TCR gd pan IMMU510 Beckman Coulter IM1349 AB_131619

TCR Vd1 TS8.2 FITC Invitrogen TCR2730 AB_223624
TCR Vd2 B6 PE BioLegend 331408 AB_1089232
TCR Vd2 B6 APC/Cy7 BioLegend 331439 AB_2860864
TCR Vg9 B3 FITC BioLegend 331306 AB_1236403
TCR Vg9 B3 PE-Cy7 BioLegend 331320 AB_2814209
Tigit A15153G Alexa Fluor 647 BioLegend 372724 AB_2715972
TIM3 7D3 BV605 BD OptiBuild 742856 AB_2744030
Viability stain 510 BD Horizon 564406 AB_2869572
Viability stain 700 BD Horizon 564997 AB_2869637

Validation

All the antibodies used in this study are commonly used for research in immunology and/or microscopy. They are well described and have been validated in previous publications or by the vendor. See for example
anti-Granzyme B 496B from Invitrogen: https://www.thermofisher.com/antibody/product/Granzyme-B-Antibody-clone-496B-Monoclonal/14-8889-82 with validation and references.
anti-Granzyme B GB11 from BD Biosciences: https://www.bdbiosciences.com/en-eu/products/reagents/flow-cytometry-reagents/research-reagents/single-color-antibodies-ruo/alexa-fluor-647-mouse-anti-human-granzyme-b.561999 used by experts in microscopy of lytic granules: https://doi.org/10.1073/pnas.2010274117
anti-CD3 UCHT1 from BD Biosciences: https://www.bdbiosciences.com/en-us/products/reagents/flow-cytometry-reagents/research-reagents/single-color-antibodies-ruo/alexa-fluor-700-mouse-anti-human-cd3.557943 and its references.
anti-perforin dG9 from BioLegend: https://www.biolegend.com/en-us/products/pacific-blue-anti-human-perforin-antibody-7265 with validation and multiple citations.
anti-CD107a H4A3 from BD Biosciences: https://www.bdbiosciences.com/en-ca/products/reagents/flow-cytometry-reagents/research-reagents/single-color-antibodies-ruo/biotin-mouse-anti-human-cd107a.555799 with multiple citations
anti-panTCRgd IMMU510 from Beckman Coulter: https://www.beckman.com/reagents/coulter-flow-cytometry/antibodies-and-kits/single-color-antibodies/tcr-pan-g-d/im1349 with validation and multiple citations.
There are no gifted antibodies in this study.

Eukaryotic cell lines

Policy information about cell lines and Sex and Gender in Research

Cell line source(s)

Main cell line: A498, ATCC, HTB-44™

In extended data figure 3c:
DLD1, ATCC
HeLa, ATCC
Daudi, ATCC
K562, ATCC
U251, provided by collaborators
Kazumi, provided by collaborators

Authentication

The A498 cell line has been validated using PCR-based single-locus technology. DLD1, HeLa. Daudi and K562 were bought directly at ATCC and expanded from the original vial for the present project and therefore not further validated. U251 and Kazumi (only used in Extended Data Figure 3c) were not validated as they play a minor role in the study.

Mycoplasma contamination

All cell lines were tested negative for mycoplasma (testing performed at Eurofins).

Commonly misidentified lines
(See ICLAC register)

None

Flow Cytometry

Plots

Confirm that:

☒ The axis labels state the marker and fluorochrome used (e.g. CD4-FITC).

☒ The axis scales are clearly visible. Include numbers along axes only for bottom left plot of group (a 'group' is an analysis of identical markers).

☒ All plots are contour plots with outliers or pseudocolor plots.

☒ A numerical value for number of cells or percentage (with statistics) is provided.

Methodology

Sample preparation

Entire PBMCs or gamma delta T cells isolated from PBMC or NK cells isolated from PBMC were washed with PBS, stained with fluorophore-conjugated antibodies for 15 min at RT in the dark, washed again with PBS prior to measurement by flow cytometry.

Instrument

BD FACS Canto II.

Software

Samples measurements were collected using BD FACS Diva Software V9.0 and the data was analyzed using FlowJo (BD)

Software	version 10.7.1.
Cell population abundance	Gamma delta TCR+ cells in fresh PBMCs was measured to be from 0.25% up to 15.8% of T cells. For non-FACS-based experiments, gamma delta T cells were isolated using MACS and only isolations with more than 90% gamma delta T cell purity were used for the assays.
Gating strategy	1. Singlets were gated from the FSC-A/FSC-H plot as the population with a linear signal correlation. 2. Lymphocytes were gated on the FSC/SSC-A plot. 3. Dead cells were excluded using a viability/death marker. 4. T cells were gated on using the marker CD3-alexa700 (population with signal over 10^3). Vg9Vd2+ T cells were gated using anti-Vg9-FITC and anti-Vd2-PE antibodies (both signals over 10^3).

☒ Tick this box to confirm that a figure exemplifying the gating strategy is provided in the Supplementary Information.
